# A study on the correlation between hyperuricemia and TG/HDL-c ratio in the Naxi ethnic group at high-altitude regions of Yunnan

**DOI:** 10.3389/fmed.2024.1416021

**Published:** 2024-08-12

**Authors:** Dongmei Han, Yaqi Yao, Fengshuang Wang, Wenjing He, Tianbao Sun, Han Li

**Affiliations:** ^1^The Rehabilitation Department of Nephrology, The First Rehabilitation Hospital of Shanghai, School of Medicine, Tongji University, Shanghai, China; ^2^Yulong County Naxi Autonomous County People's Hospital, Lijiang, Yunnan, China

**Keywords:** high altitude, hyperuricemia, triglycerides, high-density lipoprotein cholesterol, serum uric

## Abstract

**Objective:**

The study aimed to explore the risk factors for hyperuricemia (HUA) in the Naxi ethnic population residing in high-altitude areas of Yunnan, China, and assess the clinical value of the triglyceride/high-density lipoprotein cholesterol (TG/HDL-c) ratio as a diagnostic marker.

**Methods:**

In this cross-sectional study, clinical data were collected from the health checkup population in the People's Hospital of Yulong Naxi Autonomous County, Yunnan Province, from January 2021 to January 2023. Participants were divided into quartiles based on the TG/HDL-c ratio (Q1, Q2, Q3, and Q4) for group analysis using chi-square tests, *t*-tests, and rank sum tests. Logistic regression analysis and linear regression models were employed to further investigate the correlation between the prevalence of hyperuricemia and TG/HDL-c ratio in this high-altitude Naxi population.

**Results:**

A total of 714 participants from the health checkup population were included in the study, of whom 61.5% were male participants and 38.5% were female participants, and the average age was 41.21 ± 11.69 years. The mean uric acid level was 388.51 ± 99.24. After correcting for confounding factors, TG/HDL-c, serum creatinine (Scr), blood urea nitrogen (BUN), triglyceride (TG), high-density lipoprotein cholesterol (HDL-c), red blood cells (RBCs), and hemoglobin (Hb) showed a positive correlation with blood uric acid. Further analysis involved categorizing the TG/HDL-c ratio from a continuous variable to a categorical variable using quartiles. The fully adjusted model showed results that were consistent with the trend observed in the continuous variable analysis when considering the TG/HDL-c ratio as a categorical variable. In addition, in all unadjusted and adjusted models, the serum uric acid (SUA) levels in the high TG/HDL-c ratio group were significantly higher than those in the low TG/HDL-c ratio group (trend *p* < 0.001). Further linear relationship analysis indicated that after adjusting for covariates, there was an approximate linear relationship between the TG/HDL-c and SUA levels, with a coefficient (β) of 5.421.

**Conclusion:**

The prevalence of hyperuricemia is greater in high-altitude areas of Yunnan, showing a nearly linear positive correlation with the TG/HDL-c ratio. Monitoring TG/HDL-c levels may benefit patients with hyperuricemia.

## Introduction

Hyperuricemia (HUA), a common metabolic disorder characterized by elevated levels of uric acid (UA) in the blood, is caused by disturbances in purine metabolism (1). According to the 2023 Consensus of Multidisciplinary Experts on Hyperuricemia-related Diseases in China, the overall prevalence of HUA among adult residents in China from 2018 to 2019 was 14%. The prevalence was higher in male residents at 24.4% and lower in female residents at 3.6% (2). HUA prevalence varies across regions and ethnicities. Reports indicate that in the eastern and coastal regions of China, the prevalence of HUA among the population ranges from 18.78% to 44.31% in the male population and from 6.26% to 25.89% in the female population (3). In high-altitude areas, such as Qinghai and Yunnan (25.91%), the prevalence of HUA is greater than that in other regions (4). This may be attributed to the dietary patterns, socioeconomic conditions, and genetics associated with these regions or ethnic groups (5–8). However, previous studies in China have focused primarily on the Han ethnic group and regions at moderate-to-low altitudes, with research on the prevalence and risk factors for the development of HUA among minority ethnic groups in high-altitude areas, such as the Naxi ethnic group, being limited. If HUA is not effectively managed and prevented, it can lead to various complications. Relevant studies have shown that HUA status is an independent risk factor for chronic kidney disease, cardiovascular diseases, hypertension, and metabolic syndrome (9–13). The presence of HUA not only increases healthcare costs but also severely impacts the quality of life of affected individuals. Therefore, it is crucial to pay more attention to the prevalence of HUA among local minority ethnic groups and identify the risk factors associated with the development of HUA. Early and effective interventions can improve prognosis, reduce healthcare costs, and increase the overall wellbeing of the population (14).

Abnormal blood lipid concentrations are independent risk factors for the development of HUA, with hypertriglyceridemia and mixed hyperlipidemia status showing a positive correlation with HUA occurrence (15). The triglyceride-to-high-density lipoprotein cholesterol (TG/HDL-c) ratio is an emerging marker of lipid abnormalities, providing a reliable indicator for insulin resistance and metabolic syndrome (5, 6, 13). The TG/HDL-c ratio has also been demonstrated to have considerable clinical value in predicting the onset of metabolic syndrome, cardiovascular events, and chronic kidney failure (16). However, the association between the TG/HDL-c ratio and the risk of developing HUA remains unclear. In this study, we aimed to analyze the health examination data of the Naxi ethnic group residing in high-altitude areas of Yunnan Province. The objective of this study was to explore the risk factors for the development of HUA among this high-altitude minority ethnic group and investigate the correlation between the TG/HDL-c ratio and HUA occurrence. These findings provide valuable insights for the auxiliary diagnosis and prevention of HUA in the local population.

## Clinical and biochemical measurements

General information and laboratory test results were collected from the participants, including sex, age, fasting blood glucose (GLU), serum uric acid (SUA), blood creatinine (serum creatinine, Scr), blood urea nitrogen (BUN), triglyceride (TG), cholesterol (TC), high-density lipoprotein (HDL-c), low-density lipoprotein (LDL-c), white blood cells (WBCs), red blood cells (RBCs), hemoglobin (Hb), and platelets (PLTs). All participants were uniformly made to fast at 22:00 h. Blood was collected the next morning for the analysis.

The TG/HDL-c ratio was calculated by dividing the TG level by the HDL-c level, and the TG/HDL-c ratio was grouped according to the interquartile range (IQR), which converted TG/HDL-c from a continuous variable to a categorical variable (Q1, Q2, Q3, and Q4). The diagnostic criterion for hyperuricemia (HUA) was an SUA level above 420 μmol/L, regardless of gender.

### Statistical analysis

All analyses were performed using SPSS 14.0 statistical software. Measurement information with normal distribution was expressed as mean ± standard deviation. Comparisons between the two groups were made using an independent samples *t*-test, and comparisons between multiple groups were made using one-way ANOVA. Measurement information with non-normal distribution was expressed as median (lower quartile and upper quartile), and comparisons between the groups were made using the rank-sum test. Count data were expressed as the number of cases (percentage), and comparisons between the groups were made using the chi-square test. We used univariate and multivariate logistic regression analyses and univariate and multivariate linear regression models to analyze the relationship between the TG/HDL-c ratio and serum uric acid levels; a *p*-value of < 0.05 was considered statistically significant.

## Results

### Baseline characteristics of participants

**Figure 1:**
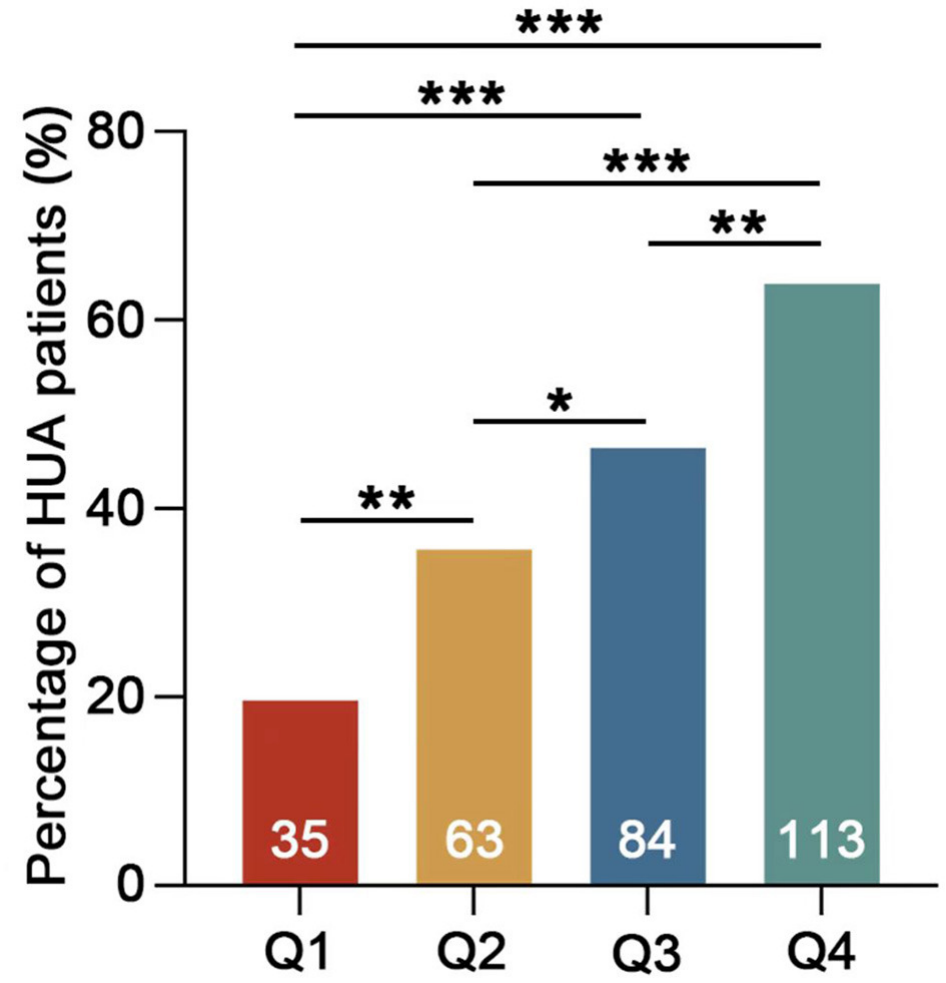
Percentage of HUA patients in different groups. Q1 TG/HDL-c <0.7 mmol/L, Q2 TG/HDL-c 0.7–1.19 mmol/L, Q3 TG/HDL-c 1.2–2.05 mmol/L, and Q4 TG/HDL-c >2.05 mmol/L. The numbers on the columns represent the number of HUA patients in each group (*p < 0.05; **p < 0.01; ***p < 0.001).

The study enrolled a total of 714 participants, of whom 61.5% were male participants and 38.5% were female participants. The average age was 41.21 ± 11.69 years, with a mean serum uric acid (SUA) level of 388.51 ± 99.24 umol/L. The overall prevalence of hyperuricemia (HUA) was found to be 41.3%. Participants were categorized into four groups based on the values of the TG/HDL-c ratio (refer to Table 1). The statistical analysis revealed significant differences in UA, triglycerides (TG), and high-density lipoprotein cholesterol (HDL-C) levels among the four groups. Specifically, the UA and TG levels exhibited a significant increase with higher TG/HDL-c ratios, while the high-density lipoprotein cholesterol (HDL-C) levels showed a significant decrease (*p* < 0.001). Further analysis demonstrated a positive association between the prevalence of HUA and increasing TG/HDL-c ratios (Figure 1). Moreover, a significant positive correlation (*p* < 0.001) was observed between the proportion of male participants, the proportion of smokers, age, and the TG/HDL-c ratio. However, no statistically significant difference was observed in blood urea nitrogen (BUN) across different TG/HDL-c groups.

**Table 1:** Baseline characteristics of the participants in Yunnan.

**Variables**	**Total**	**TG/HDL-c ratio (mmol/L) (quartiles)**				**p-value**
		**Q1 (**<**0.7)**	**Q2 (0.7–1.19)**	**Q3 (1.2–2.05)**	**Q4 (**>**2.05)**	
n	714	179	177	181	177	
Male *n*, (%)	439 (61.5)	66 (36.9)^a^	104 (58.8)^b^	125 (69.1)^b^	144 (81.4)^c^	< 0.001
Smoking *n*, (%)	324 (45.4)	47 (26.3)^a^	76 (42.9)^b^	99 (54.7)^c^	102 (57.6)^c^	< 0.001
Age (years)	41.0 (32.0–49.0)	34.0 (27.0–45.0)^a^	42.0 (32.0–49.0)^b^	45.0 (35.0–51.0)^b^	43.23 ± 11.09^b^	< 0.001
GlU (mmol/L)	5.23 (4.96–5.59)	5.01 (4.80–5.26)^a^	5.17 (4.91–5.43)^b^	5.31 (5.04–5.72)^c^	5.48 (5.17–6.17)^c^	< 0.001
UA (μmol/L)	385.55 (317.78–459.83)	317.30 (269.80–385.80)^a^	368.80 (306.65–436.80)^b^	405.00 (341.75–473.05)^c^	447.10 (381.20–508.65)^d^	< 0.001
SCR (μmol/L)	75.32 (61.74–85.98)	64.69 (58.04–80.50)^a^	74.46 (60.84–86.77)^b^	77.34 (66.64–86.55)^b^	79.65 (68.35–90.07)^bc^	< 0.001
BUN (mmol/L)	4.95 (4.15–5.77)	4.80 (4.07–5.72)	5.09 (4.22–5.89)	4.92 (4.10–5.74)	4.94 (4.26–5.78)	0.522
TG (mmol/L)	1.42 (0.97–2.25)	0.76 (0.64–0.90)^a^	1.17 (1.01–1.36)^b^	1.78 (1.57–2.00)^c^	3.10 (2.48–3.89)^d^	< 0.001
TC (mmol/L)	4.63 (4.18–5.29)	4.31 (3.88–4.72)^a^	4.59 (4.10–5.18)^b^	4.70 (4.30–5.45)^b^	5.02 (4.43–5.74)^c^	< 0.001
HDL-c (mmol/L)L	1.21 (1.02–1.41)	1.50 (1.35–1.68)^a^	1.27 (1.17–1.43)^b^	1.12 (1.01–1.26)^c^	0.98 (0.87–1.07)^d^	< 0.001
LDL-c (mmol/L)	2.70 (2.30–3.20)	2.40 (1.90–2.80)^a^	2.80 (2.30–3.20)^b^ c	3.00 (2.60–3.50)^c^	2.70 (2.10–3.40)^bd^	< 0.001
WBC(^*^109/)L	5.72 (4.79–6.88)	5.22 (4.43–6.24)^a^	5.56 (4.57–6.63)^ab^	5.90 (4.98–6.96)^bc^	6.20 (5.29–7.35)^c^	< 0.001
RBC(^*^1012/)L	5.12 (4.73–5.49)	4.77 (4.55–5.16)^a^	5.06 (4.71–5.45)^b^	5.24 (4.89–5.52)^b^	5.41 (5.05–5.69)^c^	< 0.001
Hb (g/L)	158.0 (145.0–168.25)	147.0 (140.0–159.0)^a^	155.0 (145.0–166.5)^b^	162.0 (148.5–169.5)^b^	165.0 (155.0–174.0)^c^	< 0.001
PLT (^*^109/L)	226.0 (194.0–260.25)	227.0 (193.0–260.0)	224.0 (190.0–255.0)	226.0 (198.0–266.0)	228.0 (196.5–261.0)	0.772
HUA *n*, (%)	295 (41.3	35 (19.6)^a^	63 (35.6)^b^	84 (46.4)^b^	113 (63.8)^c^	< 0.001

^*^If the letters abcd are the same between two groups, it indicates *p* > 0.05. If the letters between two groups are different, it indicates *p* < 0.05.

### Correlation between the TG/HDL-c ratio and HUA

The univariate logistics analysis, as shown in Table 2, reported that higher levels of the TG/HDL-c ratio were positively correlated with the presence of HUA [odds ratio (OR) = 1.135, 95% confidence interval (CI) 1.176–1.471, *p* < 0.001, Table 2]. In addition, male, smoking, and high levels of Scr, BUN, TG, HDL-c, RBC, and Hb were also risk factors for HUA (*p* < 0.001, Table 2), while factors including age, Glu, TC, and PLT, did not show any significant relationship with the development of HUA.

**Table 2:** Univariate logistics regression analysis of HUA in the Yunnan region.

**Variables**	**Statistics of HUA**	**Coefficient**	**Standard Error**	**OR (95%CI)**	***p*-value**	**Hosmer–Lemeshow**	
						**Chi-square**	**Significance**
TG/HDL-c	1.69 (0.97–2.61)	0.274	0.047	1.315 (1.176–1.471)	< 0.001	38.392	< 0.001
Male *n*, (%)	251 (57.2)	1.947	0.191	7.009 (4.824–10.186)	< 0.001	0	/
Smoking *n*, (%)	180 (55.6)	1.095	0.158	2.989 (2.195–4.071)	< 0.001	0	/
Age (years)	41.0 (32.0–49.0)	0.003	0.006	1.003 (0.990–1.016)	0.694	4.062	0.852
Glu (mmol/L)	5.30 (5.04–5.74)	0.058	0.051	1.060 (0.960–1.171)	0.251	22.306	0.004
Scr (μmol/L)	83.01 (74.23–91.40)	0.063	0.006	1.066 (1.053–1.078)	* < 0.001*	22.02	0.005
BUN (mmol/L)	5.24 (4.40–6.01)	0.291	0.063	1.338 (1.182–1.515)	< 0.001	9.746	0.283
TG (mmol/L)	1.84 (1.20–2.66)	0.298	0.063	1.347 (1.191–1.523)	< 0.001	32.244	< 0.001
TC (mmol/L)	4.72 (4.25–5.39)	0.001	0.039	1.001 (0.928–1.080)	0.979	6.66	0.574
HDL-c (mmol/L)	1.12 (0.97–1.27)	−2.188	0.306	0.112 (0.062–0.204)	< 0.001	9.915	0.271
LDL-c (mmol/L)	2.80 (2.30–3.20)	0.195	0.097	1.216 (1.005–1.471)	0.045	4.69	0.79
WBC (^*^10^9^/)L	5.91 (5.00–7.05)	0.119	0.046	1.126 (1.029–1.233)	0.01	9.382	0.311
RBC (^*^10^12^/)L	5.34 (5.05–5.61)	1.206	0.164	3.340 (2.423–4.603)	< 0.001	17.92	0.022
Hb (g/L)	164.0 (155.0–164.0)	0.044	0.006	1.045 (1.034–1.057)	< 0.001	16.639	0.034
PLT (^*^10^9^/L)	222.0 (192.0–254.0)	−0.002	0.001	0.998 (0.995–1.001)	0.109	5.139	0.743

### Linear relationship between TG/HDL-c and serum uric acid

The linear relationship analysis performed revealed that there was an approximate linear relationship between the TG/HDL-c and serum UA levels, after controlling for sex, smoking status, SCr, BUN, LDL-c, WBC, and RBC [β = 5.421, 95% confidence interval (CI) 3.368–7.473, *p* < 0.001]. It is important to note that there was only a slight presence of multicollinearity between the TG/HDL-c and serum UA levels (VIF = 1.08; Table 3).

**Table 3:** Linear model analysis.

**Model**	**Unstandardized Coefficients**		***t***	**Significance**	**Confidence Interval 95%**		
	**B**	**Standard Error**			**Lower Limit**	**Upper Limit**	**Tolerance**
TG/HDL-c	5.421	1.045	5.186	< 0.001	3.368	7.473	0.926

Adjusted for sex, smoking, SCr, BUN, LDL-c, WBC and RBC.

### Incidence of HUA according to TG/HDL-c

We conducted logistic regression analysis using both unadjusted and adjusted models to further demonstrate that the TG/HDL-c ratio is an independent predictor of elevated serum UA levels (Table 4). In the unadjusted model, the TG/HDL-c ratio was positively correlated with the incidence of HUA [OR = 1.315, 95% confidence interval (CI): 1.176–1.471, *p* < 0.001]. In the fully adjusted model (adjusting for sex, smoking status, SCr, BUN, LDL-c, WBC, and RBC), a higher TG/HDL-c ratio was still positively correlated with the prevalence of HUA (OR=1.199, 95% CI: 1.077–1.334, *p* < 0.001). The model showed a high level of fitting *p* = 0.697.

**Table 4:** The relationship between the TG/HDL-c ratio and HUA in different models.

**Variables**	**Non-adjusted model**			**Fully adjusted model**		
	**Coefficient (Standard Error)**	**OR (95%CI)**, ***p*****-value**	**Hosmer–Lemeshow test** ***p*****-value**	**Coefficient (Standard Error)**	**OR (95%CI)**, ***p*****-value**	**Hosmer–Lemeshow test** ***p*****-value**
TG/HDL-c	0.274 (0.047)	1.315 (1.176–1.471), < 0.001	< 0.001	0.181 (0.055)	1.199 (1.077–1.334), 0.001	0.697
**TG/HDL-c group**						
Q1		Ref	**/**		Ref	0.124
Q2	0.712 (0.216)	2.039 (1.335–3.114), 0.001		0.612 (0.239)	1.843 (1.154–2.944), 0.010	
Q3	1.162 (0.222)	3.195 (2.069–4.933), < 0.001		0.980 (0.247)	2.665 (1.641–4.327), < 0.001	
Q4	1.983 (0.245)	7.264 (4.495–11.740), < 0.001		1.555 (0.281)	4.735 (2.729–8.213), < 0.001	
*p* for trend		< 0.001			< 0.001	

We also converted the TG/HDL-c ratio from a continuous variable to a categorical variable (quartiles), the *p*-value for the trend of the categorized TG/HDL-c ratio in the fully adjusted model matched with the result when the TG/HDL-c ratio was a continuous variable. We found significantly higher serum UA levels in the middle and high TG/HDL-c ratio (Q2-Q4) groups compared with the low TG/HDL-c ratio (Q1) group among all the unadjusted and adjusted models (the *p-*value for the trend was < 0.001). The model showed a high level of fitting (*p* = 0.124). This suggested a linear relationship between the levels of the TG/HDL-c ratio and HUA.

## Discussion

HUA is considered a common and prevalent condition in high-altitude regions, and previous studies have shown a greater incidence of HUA in high-altitude areas than in plains areas (3). Monosodium urate (MSU) deposition in the joints of patients can lead to gouty arthritis and joint deformity. If urate salts are deposited in the kidneys, it may result in UA nephropathy and urolithiasis, eventually leading to renal failure. Urate salts stimulating vascular walls can contribute to the development of atherosclerosis, exacerbating the risk of developing hypertension and coronary heart disease. The present study revealed the total prevalence of HUA to be 41.31% in the Naxi ethnic group in Yunnan, China. The HUA prevalence was 57.17% in male residents and 16% in female residents, which was significantly higher than the 14% prevalence of HUA reported in domestic studies. The higher incidence of HUA in high-altitude regions can be attributed to various factors. Previous studies have demonstrated that in the hypoxic environment of high-altitude areas, the reduced degradation of hypoxanthine leads to the accumulation of xanthine intermediates (17, 18). This, in turn, increases the formation of purine metabolites. In addition, in a hypoxic environment, increased concentrations of lactate in the body exert an inhibitory effect on UA excretion (19, 20). Furthermore, hypoxia induces an increase in the production of RBCs and accelerates the synthesis and breakdown of hemoglobin, resulting in elevated levels of purine metabolites and endogenous cellular breakdown products, thereby further contributing to the elevation of serum UA (SUA) levels (21).

Currently, increasing evidence indicates the significant impact of smoking status on SUA levels worldwide (22–25), a phenomenon that was also observed in our study. Epidemiological studies have suggested a negative correlation between smoking status and SUA levels, which can be attributed to oxidative stress resulting from long-term exposure to smoking (26–28). In addition, SUA levels may be influenced by genetic factors, occupational factors, and unhealthy lifestyle habits (29, 30). Notably, the identification of specific genes associated with rare monogenic disorders that lead to markedly elevated UA levels provides insights into the etiology of HUA. For example, familial juvenile hyperuricemia nephropathy is caused by deficiencies in hypoxanthine-guanine phosphoribosyltransferase or the overactivity of phosphoribosyl pyrophosphate synthetase-1 (31, 32). Moreover, the prevalence of HUA is closely associated with local temperature, barometric pressure, and air humidity. The Lijiang region in Yunnan is a high-altitude area characterized by high barometric pressure and low air density. Under these conditions, urate salts in the joint cavities of individuals with HUA are more prone to form deposits. Diet plays a significant role in HUA, as an increased intake of purine-rich foods can lead to elevated UA production (33, 34). Previous studies have indicated that a plant-based diet, a Mediterranean diet, and a low-sodium diet are associated with a decreased risk of developing HUA (35, 36). Furthermore, previous studies have shown that the consumption of meat and poultry can increase UA levels (37). Conversely, the consumption of dietary antioxidants, such as dietary fiber, zinc, magnesium, and vitamin D, has been shown to inhibit the development of HUA (38–41). Further analysis of the dietary patterns of the Naxi ethnic group in the Lijiang region of Yunnan revealed that the majority of the population consists of ethnic minorities who consume a high-sodium diet. They tend to consume beef, mutton, barley, and butter tea, and they prefer cured meats, such as cured pork ribs, three-line meat, and sausages, as well as ham. There is also a higher prevalence of alcohol and tobacco consumption among the population. These findings provide valuable insights for further research on the associations between dietary patterns, lifestyle habits, and the incidence of HUA. These findings also enable the local healthcare system to implement and improve health screenings in the region and develop interventions in terms of nutrition education and lifestyle modifications (42).

The correlations of the TG/HDL-c ratio and the concentrations of SCr, BUN, TG, HDL-c, RBC, and Hb with the UA levels were assessed. In the fully adjusted model presented in Table 3, we further considered the influence of variables, such as sex, smoking status, the concentrations of SCr, BUN, LDL-c, and the WBC and RBC counts. The results shown in Table 3 indicate that, in the adjusted model, the TG/HDL-c ratio remained positively associated with the SUA levels. Specifically, there was an approximately linear relationship with a coefficient of 5.421 [95% confidence interval (CI): 3.368–7.473, *p* < 0.001]. For each one-unit increase in the TG/HDL-c ratio value, the average SUA level increased by 5.421 μmol/L. This finding indicates that the TG/HDL-c ratio is an independent factor influencing the risk of developing HUA and is not influenced by other potential factors. The TG/HDL-c ratio reflects the disruption of lipid metabolism and abnormal metabolism of cholesterol and TGs, which can lead to abnormal production and excretion of UA. As a result, UA accumulates, thereby increasing the risk of developing HUA and the risk of its progression. The mechanism behind this may be that when the body produces more lipids than it can metabolize, lipids accumulate in organs, such as the kidneys. Excessive lipid deposition can trigger various signaling pathways, including inflammation, oxidative stress, and autophagy, leading to the development of various diseases. In addition, lipid deposition can promote the proliferation of glomerular basement membrane cells, exacerbate glomerulosclerosis, damage the renal tubular interstitium, and reduce the excretion of UA (43, 44), significantly increasing the risk of developing HUA. Therefore, the TG/HDL-c ratio may also reflect the extent of chronic inflammation, which, in turn, affects the metabolism and excretion of UA (45–48), leading to the occurrence and progression of HUA. Therefore, the determination of the TG/HDL-c ratio is not only useful for evaluating an individual's lipid status and degree of UA metabolism disorder but also has reference value for early screening of HUA in high-altitude areas (49). This finding is consistent with our research, indicating that the TG/HDL-c ratio has significant implications for the diagnosis and treatment of HUA in high-altitude regions.

Several previous studies (50–54) have shown a positive correlation between C-reactive protein (CRP) and the prevalence of HUA or high UA levels. However, another study demonstrated that elevated UA levels were not associated with CRP concentrations. Additionally, a previous study by Nigerian researchers revealed no associations between the SUA concentration and the erythrocyte sedimentation rate (ESR), CRP concentration, or WBC count. Furthermore, a study conducted in China also indicated no association between the SUA concentration and the CRP concentration or WBC count (55). We believe that an elevated UA concentration in patients with gouty arthritis may increase the WBC count, the concentrations of inflammatory markers, the ESR, and the CRP concentration. However, the participants in our study were individuals from a healthy population undergoing routine medical checkups, and their UA concentrations had not reached levels that would induce elevation of inflammatory markers or increased WBC counts. They did not exhibit any signs of acute inflammation, such as an elevated WBC count, ESR, or hs-CRP concentration. This finding provides a good direction for further investigations into the relationships of HUA status with the risk of developing gout, the WBC count, the ESR, and hs-CRP concentrations.

Although the results of this study provide valuable insights, there are still several limitations. First, owing to the cross-sectional design of the study, causal relationships could not be determined. Therefore, long-term longitudinal studies are needed to validate the impact of the TG/HDL-c ratio on the risk of developing HUA and the predictive value of the TG/HDL-c ratio. Second, the study sample included only middle-aged and elderly individuals from high-altitude areas in Yunnan, and it was a single-center study. In the future, we will increase the sample size, use a multicenter design, and improve follow-up to confirm these research findings. Finally, in this study, we did not consider other important potential confounding factors, such as dietary habits, lifestyle factors, genetic factors, body mass index, and physical activity levels. These factors may influence the relationship between the TG/HDL-c ratio and SUA levels.

Overall, our research results support the correlation between the TG/HDL-c ratio and the risk of developing HUA in high-altitude areas among the Naxi ethnic group. An increased TG/HDL-c ratio may be associated with an increased risk of developing HUA, providing a reliable biomarker for the prevention, diagnosis, and treatment of HUA.

## Conclusion

The TG/HDL-C ratio was significantly and positively correlated with SUA.

## Data availability statement

The original contributions presented in the study are included in the article/supplementary material, further inquiries can be directed to the corresponding authors.

## Ethics statement

The studies involving humans were approved by the Ethics Committee of Shanghai First Rehabilitation Hospital. The studies were conducted in accordance with the local legislation and institutional requirements. The participants provided their written informed consent to participate in this study.

## Author contributions

DH: Writing – original draft, Writing – review & editing, Data curation, Formal analysis, Investigation. YY: Writing – original draft, Writing – review & editing. FW: Writing – review & editing, Investigation. WH: Writing – review & editing, Data curation. TS: Supervision, Writing – review & editing. HL: Funding acquisition, Writing – original draft, Writing – review & editing.
